# Exploring Inflammatory Markers and Risk Factors Associated with Pericarditis Development after Ablation for Atrial Fibrillation

**DOI:** 10.3390/jcm13195934

**Published:** 2024-10-05

**Authors:** Idris Yakut, Hasan Can Konte, Ozcan Ozeke

**Affiliations:** 1 Department of Cardiology, Medipol Istanbul University, Istanbul 34815, Türkiye; hcankonte@gmail.com; 2 Department of Cardiology, Health Sciences University Ankara City Hospital, Ankara 06800, Türkiye; ozcanozeke@gmail.com

**Keywords:** atrial fibrillation, ablation techniques, pericarditis, inflammatory markers, risk factors

## Abstract

**Background:** This study aimed to explore the association between inflammatory markers and the occurrence of post-atrial fibrillation (AF) ablation pericarditis (PAP), while also examining the PAP’s incidence and contributing factors. **Methods:** A retrospective cohort study was conducted between January 2021 and November 2023, including patients who underwent successful AF ablation. Inflammatory markers of interest included the systemic immune-inflammation index (SII), the neutrophil-to-lymphocyte ratio (NLR), and the platelet-to-lymphocyte ratio (PLR). **Results:** Among the 231 patients examined, 22 (9.52%) were classified as suspected PAP, and 14 (6.06%) as definitive PAP. The median age was 58 years, with no age difference between groups. Males comprised 51.52% of the sample, with male sex frequency significantly higher in the suspected PAP group relative to the other groups (*p* = 0.007). Multivariable logistic regression indicated that AF duration (*p* = 0.026) and cavotricuspid isthmus (CTI) ablation (*p* = 0.001) were associated with definitive PAP, whereas analysis for any pericarditis (suspected or definitive PAP) revealed independent relationships with CTI ablation (*p* = 0.003) and sleep apnea (*p* = 0.008). SII, NLR, and PLR were not associated with PAP. **Conclusions:** Prolonged AF duration, CTI ablation, and sleep apnea are risk factors for PAP. The inflammatory markers (SII, NLR, and PLR) showed no association, warranting further investigation into other markers.

## 1. Introduction

Atrial fibrillation (AF) is the most prevalent form of sustained cardiac arrhythmia, and it burdens the healthcare system with increased costs as well as causing morbidity and poor quality of life [1]. Catheter ablation is a cornerstone in the management of symptomatic, drug-refractory AF, potentially restoring the cardiac rhythm [2]. However, AF ablation can cause complications, one of which is pericarditis, an inflammatory condition of the pericardium that can lead to a range of clinical consequences [2,3].

Pericarditis following AF ablation (post-ablation pericarditis—PAP) is an important but often overlooked complication [4]. Traditional thermal ablation modalities, such as the cryoballoon or radiofrequency (RF) methods, can cause acute pericarditis in 0.8–10.2% of recipients [4,5]. The acute pericarditis in some patients may even progress into more severe conditions such as pericardial effusion, tamponade, or constrictive pericarditis [3]. The development of PAP due to ablation is attributed to (or associated with) several factors, including direct thermal injury to the pericardium, inflammatory overactivation in response to tissue damage, and the exacerbation of underlying pericardial pathology [6]. Despite the importance of PAP, its reported incidences vary widely from study to study, and there is a lack of consensus regarding predisposing risk factors [3].

Inflammation is believed to play a significant role in the pathogenesis of PAP since the ablation process induces local tissue damage, which gives rise to inflammation within the pericardium [7]. The identification of markers that predict or correlate with the risk of developing PAP is crucial for improving patient outcomes and guiding post-procedural management. The systemic immune-inflammation index (SII), the neutrophil-to-lymphocyte ratio (NLR), and the platelet-to-lymphocyte ratio (PLR) have emerged as easily accessible markers that reflect inflammatory activation and sometimes immune status in various diseases [8,9]. However, their specific relationship with the development of PAP remains underexplored.

This study aims to investigate the association between these inflammatory markers—SII, NLR, and PLR—and the occurrence of PAP, while also attempting to explore the PAP incidence and other risk factors associated with its development.

## 2. Materials and Methods 

### 2.1. Study Design, Participants, and Ethical Issues

This was a retrospective cohort study conducted between January 2021 and November 2023, involving patients who underwent successful AF ablation procedures at the Cardiology Clinics of Ankara City Hospital in Ankara, Turkey, and Medipol University Bahçelievler Medipol Hospital in Istanbul, Turkey. All the eligible patients were included in the analyses unless they had developed procedural pericardial complications due to the ablation procedure or had incomplete or missing data. This study was performed in line with the principles of the Declaration of Helsinki. Approval was granted by the Non-Interventional Clinical Research Ethics Committee of Istanbul Medipol University (decision date: 4 January 2024, decision number: 18).

### 2.2. Data Collection and Definitions

#### 2.2.1. Clinical and Laboratory Data

The patients were classified as paroxysmal, persistent, or long persistent AF according to the duration and nature of the AF [10]. The duration of AF in patients was also included as a separate variable in this study.

The medical records of the patients who underwent AF ablation were retrospectively collected from computerized records. This study examined information on age, sex, body mass index (BMI), and comorbidity; smoking status (non-smoker, ex-smoker, active smoker); AF-related features (AF duration, and medication data including oral anticoagulants, antiarrhythmic agents, and statin use); echocardiographic measurements (left-ventricular ejection fraction and left-atrial diameter); laboratory findings including creatinine, hemoglobin, glucose, albumin, total cholesterol, high-density lipoprotein cholesterol (HDL-C), low-density lipoprotein cholesterol (LDL-C), triglyceride, gamma glutamyl transferase (GGT) and C-reactive protein (CRP) levels, and white blood cell, neutrophil, lymphocyte, and platelet counts.

#### 2.2.2. Ablation Data

We also examined the AF ablation-related features in detail. The AF ablation-related features included whether the patient underwent a redo ablation and the type of ablation (RF or cryoablation). For RF, the number of applications, the average RF ablation duration, the anterior and posterior wattage applied, and the presence of atrial scar tissue resulting from the procedure were among the parameters examined. For cryoablation, we extracted the duration of each cryoablation application, the peak cryoablation temperature, and the number of applications. Additionally, specific ablation sites and techniques were recorded, including cavotricuspid isthmus (CTI) ablation, ablation lines (anterior mitral, roof, or anterior mitral + roof), posterior-wall ablation, and focal ablation.

#### 2.2.3. Pericarditis Data

The pericarditis-related features included pericardial chest pain, electrocardiography (ECG) findings, pericardial effusion, pericardial rubs, and fever. Treatment- and management-related data were also recorded, including medications (no treatment, ibuprofen, colchicine, colchicine + ibuprofen).

#### 2.2.4. CHA2DS2-VASc Score

The CHA2DS2-VASc scores were calculated for each patient using baseline demographic and echocardiographic data. According to the CHA2DS2-VASc scoring system, patients were assigned 1 point each for the presence of congestive heart failure, hypertension, being aged 65–74 years, diabetes, vascular disease, and female sex. Two points were assigned for being ≥75 years of age and having a history of stroke or transient ischemic attack [11].

### 2.3. Ablation Procedures

Prior to the ablation procedure, arrythmias were closely managed using antiarrhythmics. On the day of the procedure, local anesthesia and sedation were administered, and a catheter was introduced through the femoral vein.

For radiofrequency ablation, the Carto three-dimensional electroanatomic mapping system was employed to precisely map the pulmonary veins. An RF ablation catheter (ThermoCool SmartTouch, Biosense Webster, Irvine, CA, USA) was used to deliver RF energy to the heart and disable abnormal electrical circuits within the heart.

In cases in which cryoablation was performed, a cryoballoon ablation catheter (Arctic Front Advance, AFA-Pro, Medtronic, Minneapolis, MN, USA) was used to map and isolate the pulmonary veins. The catheter tip was cooled to −50 °C to −70 °C to permanently disable AF-inducing circuitry.

After the procedure, the patients were closely monitored in a cardiac observation unit for at least 24 h to identify any potential complications. If the patients had symptoms following ablation, 24 h ECG Holter monitoring was employed to detect AF recurrence. The routine follow-up involved regular clinical evaluations and ECG rhythm monitoring throughout the recovery phase, with scheduled cardiology outpatient visits at 1, 3, and 12 months after the procedure.

### 2.4. Post-Ablation Pericarditis Management

The diagnosis and treatment of pericarditis were guided by the definitions outlined in the European Society of Cardiology (ESC) pericarditis guidelines [12]. According to the ESC criteria, a diagnosis of pericarditis requires the presence of at least two of the following findings within 3 months after the ablation procedure: pericardial chest pain, a new friction rub, new or worsening effusion, new widespread ST elevation, or PR depression on the ECG [12]. Suspected acute pericarditis after AF ablation was defined as pericardial chest pain treated with anti-inflammatory medications (ibuprofen, colchicine) either at hospital discharge or within three months after AF ablation. Pericardial chest pain was characterized as sharp and pleuritic, typically improving with sitting up and leaning forward [3].

### 2.5. Laboratory Analysis

Routine laboratory findings obtained from venous blood samples taken before the AF ablation procedure were included as variables in this study. The laboratory analyses were performed at the certified biochemistry laboratories of both study centers. The NLR was calculated by dividing the absolute neutrophil count by the absolute lymphocyte count. The PLR was determined by dividing the absolute platelet count by the absolute lymphocyte count. Additionally, the SII was computed by multiplying the platelet count (10^9^/L) by the neutrophil count (10^9^/L) and then dividing the result by the lymphocyte count (10^9^/L) [9,13]. 

### 2.6. Statistical Analysis

According to descriptive statistics (effect size = 0.380) in the study by Yilmaz Y. et al. [14], a total sample size of 72 achieves 80% power at the two-sided 0.05 significance level. The sample size was calculated using a one-way analysis of variance power analysis (Hintze, J. (2011). PASS 11. NCSS, LLC. Kaysville, UT, USA, www.ncss.com accessed on 8 August 2024).

All analyses, subject to a *p* value threshold of <0.05 for significance, were performed on SPSS Statistics v25 (IBM Corp., Armonk, NY, USA). For the normality check, histograms and Q-Q plots were used. Descriptive statistics were presented using mean ± standard deviation for normally distributed continuous variables and median (25th percentile–75th percentile) for non-normally distributed continuous variables, while frequency (percentage) was used for categorical variables. The normally distributed variables were analyzed with one-way analysis of variance (ANOVA). The non-normally distributed variables were analyzed with the Kruskal–Wallis test. The categorical variables were analyzed with the chi-square test or the Freeman–Halton extension test. Pairwise comparisons were adjusted using the Bonferroni correction. Multivariable logistic regression (forward conditional selection) was performed to determine significant factors independently associated with pericarditis. The multivariable models inherited variables that were significant according to univariate analyses. 

## 3. Results

**Figure 1:**
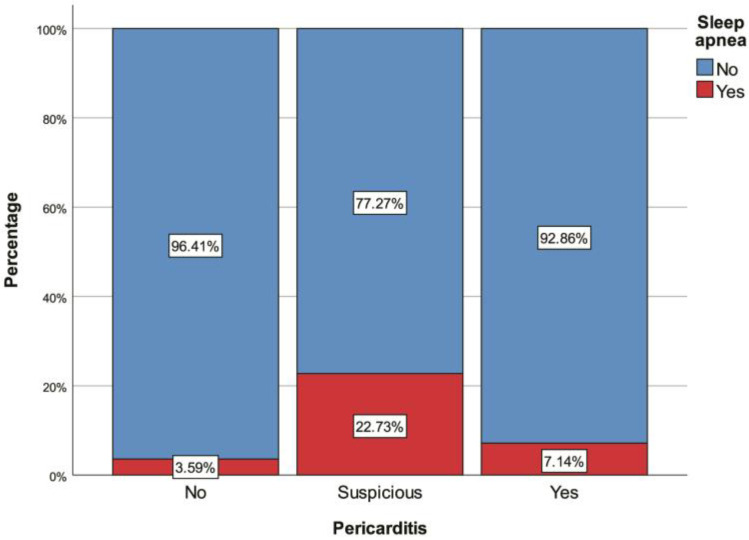
Sleep apnea percentages with regard to pericarditis.

**Figure 2:**
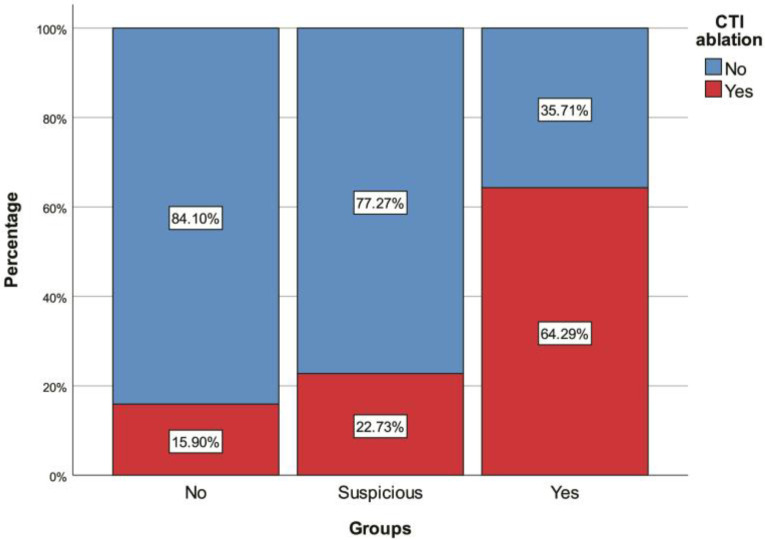
Box plots of duration of atrial fibrillation (AF) with regard to pericarditis.

**Figure 3:**
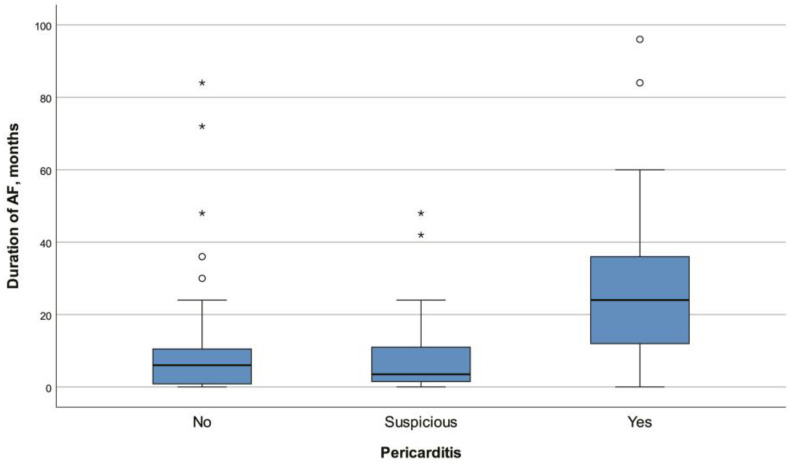
CTI ablation percentages with regard to pericarditis. Circle and asterisk representing outliers and extreme outliers, respectively.

A total of 231 patients were included in this study. Twenty-two (9.52%) were in the suspicious PAP group, and fourteen (6.06%) were in the definitive PAP group. The median age of all the patients was 58 (48–66) years. There was no significant difference in age between the three groups (non-PAP vs. definitive PAP vs. suspected PAP; *p* = 0.692). Of all the patients, 51.52% (*n* = 119) were male. The percentage of males in the suspected PAP group was significantly higher than in the other two groups (*p* = 0.007). Sleep apnea was also significantly more common in the suspected PAP group than in the non-PAP group (*p* = 0.004) (Figure 1). The definitive PAP group demonstrated significantly higher frequencies of paroxysmal AF (*p* = 0.007), RF ablation (*p* = 0.016), CTI ablation (*p* < 0.001) (Figure 2), and receiving colchicine monotherapy for pericarditis treatment (*p* < 0.001) relative to both other groups. Furthermore, the AF duration (*p* < 0.001) (Figure 3), left-atrium diameter (*p* = 0.008), lymphocyte count (*p* = 0.038), and median number of RF applications (*p* = 0.007) were also significantly higher among patients with definitive PAP than in the non-PAP and suspected PAP groups (Table 1, Table 2, Table 3 and Table 4).

When focusing on the definitive PAP versus non-PAP comparison, we found longer RF duration (*p* = 0.013) and higher frequencies of long persistent AF (*p* = 0.007), redo ablation (*p* = 0.020), and posterior-wall ablation (*p* = 0.004) in the definitive PAP group than in the non-PAP group. Additionally, the definitive PAP group had significantly lower anterior watt (*p* = 0.015) and posterior watt (*p* = 0.035) values than the non-PAP group. The median platelet count in the definitive PAP group was significantly higher than in the suspected PAP group (*p* = 0.028). The percentage of patients receiving ibuprofen monotherapy in the suspicious PAP group was significantly higher than in the definitive PAP group (*p* < 0.001) (Table 1, Table 2, Table 3 and Table 4).

The parameters that had an independent association with definite PAP were identified as longer AF duration (OR: 1.017, 95% CI: 1.002–1.032, *p* = 0.026) and having undergone CTI ablation (OR: 7.812, 95% CI: 2.404–25.386, *p* = 0.001), based on multivariable logistic regression. The other variables included in the model, sex (*p* = 0.235), sleep apnea (*p* = 0.599), long persistent AF (*p* = 0.865), left-atrium diameter (*p* = 0.361), lymphocyte (*p* = 0.093), platelet (*p* = 0.204), redo ablation (*p* = 0.885), type of ablation (*p* = 0.247), and posterior-wall ablation (*p* = 0.410), were found to be non-significant (Table 5).

The multivariable logistic regression analysis for any type of PAP diagnosis (definite + suspected) revealed that sleep apnea (OR: 5.062, 95% CI: 1.529–16.765, *p* = 0.008) and CTI ablation (OR: 3.256, 95% CI: 1.476–7.186, *p* = 0.003) were independently associated with the presence of PAP. The other variables included in the analysis, sex (*p* = 0.192), long persistent AF (*p* = 0.666), duration of AF (*p* = 0.419), left-atrium diameter (*p* = 0.303), lymphocyte (*p* = 0.465), platelet (*p* = 0.356), redo ablation (*p* = 0.127), type of ablation (*p* = 0.850), and posterior-wall ablation (*p* = 0.156), were found to be non-significant (Table 6).

## 4. Discussion

In this study, definite PAP was diagnosed in 6.06% of patients, while the overall PAP (definite and suspected) frequency was 15.6%. The inflammatory parameters examined (SII, NLR, and PLR) were not found to be associated with the development of PAP. The independent risk factors for the occurrence of definite PAP were CTI ablation and longer AF duration. When considering both definite and suspected PAP, the presence of sleep apnea and the CTI ablation were identified as the independent risk factors.

The indications for catheter ablation of AF have increased, and, with advanced mapping and ablation technologies, the success rate of this procedure can reach up to 90%. However, the complications associated with the procedure remain as considerable problems, both major and minor, including stroke, pulmonary vein stenosis, esophageal injury, and phrenic nerve palsy, as well as pericarditis [1,2,15]. Acute pericarditis is one of the significant complications arising from RF and cryoballoon ablation procedures due to direct myocardial tissue damage and irritation [1]. Such PAP cases have largely been reported with case reports and series, and, thus, the number of studies reporting PAP incidence has remained limited. In the present study, we report a frequency of 6.06% for definite PAP diagnosis during a study period of almost 3 years (35 months), while suspected PAP was diagnosed in an additional 9.5% of subjects, revealing an overall PAP frequency of 15.6% in our study group. PAP rates have been reported to range from 0% to 17.4% in different studies [2,3,4,5,16,17,18,19,20,21], and the frequency in our study falls within this range. This wide range is likely due to the variations in the ablation procedures used and the definitions of PAP (including classification of definite PAP, suspected PAP, or both). Therefore, comprehensive studies using homogeneous patient groups, procedure-specific approaches, and standardized definitions for PAP are needed to determine the exact incidences.

Understanding the relationship between inflammation and the development of PAP is crucial for managing and preventing this complication. The ablation procedure can trigger an inflammatory response through electrical and structural changes in the heart. Pericarditis often results from pericardial inflammation, which may develop due to trauma from the procedure, thermal effects, and tissue damage [3]. In this context, it was conceivable to presume that inflammatory markers might play a significant role in assessing the risk of pericarditis, based on prior data associating different disease manifestations with inflammation [9,22,23]. Our study primarily aimed to investigate the association between PAP development and easily accessible inflammation markers calculated from complete blood counts. However, the results showed that SII, NLR, and PLR were unassociated with PAP development. This finding may be attributed to several possible factors. Firstly, while these markers reflect inflammation and immune responses, they do not include the assessment of all pathophysiological processes involved in pericarditis development, and the systemic response may be insufficient to create detectable variations in the circulatory cell composition. Pericarditis is a complex condition resulting from both inflammatory and mechanical factors. To better understand the impact of inflammation on this specific complication, a more comprehensive examination of detailed inflammatory markers may be required. Secondly, our study design and methods might also influence the results. Factors such as the size of the study group (especially considering the small size of the positive groups) and criteria for diagnosing pericarditis may have posed limitations on determining the effect of inflammation. Thirdly, individual differences in clinical practice could also play a role, as the development of pericarditis may vary based on immune response, genetic predisposition, and other comorbid conditions [24]. Finally, the development of pericarditis may result from both systemic and local inflammation. Therefore, it might be necessary to assess specific inflammatory responses occurring in the pericardial tissue. Targeted approaches, such as pericardial fluid analysis, tissue-specific markers, and evaluation of inflammatory cells, may improve the examination of the relationships between pericarditis and inflammation. Additionally, more detailed analyses evaluating the stages and severity of inflammation could contribute to understanding the inflammatory process involved in pericarditis development [25]. The current literature on the relationship between inflammatory markers and pericarditis is limited, and to our knowledge, significant results have not yet been reported. Our findings suggest that the effects of inflammation on pericarditis development need to be studied in larger and more diverse patient populations. Understanding the effects of inflammation on pericarditis is important for developing strategies to reduce post-ablation complications. Future studies examining inflammation at both systemic and local levels may facilitate a better understanding and management of PAP.

As with the incidence of PAP, the factors that increase the risk of PAP have not been sufficiently studied. However, various factors, such as preferred ablation techniques, patient age, comorbidities, and medication use, are likely to play a role in the development of pericarditis. Technical features, particularly large ablation areas, prolonged procedure times, and consecutive ablations, may trigger pericardial inflammation [3]. Understanding PAP risk factors is crucial for several reasons. First, it could lead to improved patient selection, allowing clinicians to better identify individuals at higher risk and manage diagnostic/prognostic processes. Second, insights into these risk factors may improve procedure application and reduce the incidence of pericarditis. Lastly, prophylactic strategies may be possible in patients with very high risks, such as the early initiation of anti-inflammatory medications.

Our secondary purpose was to investigate the risk factors associated with PAP to better understand the underlying mechanisms of this complication and contribute to preventive strategies. Our results showed that undergoing CTI ablation and having prolonged AF were independent risk factors for definitive PAP. When considering all pericarditis cases (both definitive and suspected), the presence of sleep apnea and CTI ablation were identified as independent risk factors for PAP. In the limited number of studies on this topic, various risk factors for PAP have been identified. Darmoch et al. reported female sex, obesity, rheumatoid arthritis, and low hemoglobin levels as risk factors [2]. In several studies, the total freezing time and the number of cryoapplications were reported to be significantly higher in patients who developed pericarditis after cryoballoon ablation [16,26]. Interestingly, Nakhla et al. identified young age as the sole factor associated with developing suspected PAP. In their study, hypertension, high BMI, lower CHADS2-VASc scores, and redo AF were significantly associated with PAP in univariate analysis; however, none of these variables were identified as independent risk factors in multivariable analysis [3]. This was partially supported by another recent study that identified RF ablation as an independent predictor of PAP, while older age was associated with a decreased risk [5]. The type of ablation and its application have been identified to be responsible for PAP in other studies as well. For instance, a prospective study concluded that ablation with posterior-wall isolation was associated with a higher risk of pericarditis compared to pulmonary vein isolation alone [18].

Anti-inflammatory medications also appear to impact outcomes in these patients. Mohanty et al. investigated the relationship between colchicine monotherapy and PAP. Patients were divided into groups based on colchicine use: those who did not receive colchicine, those who used colchicine from 7 days before ablation until 1 month after, and those who used colchicine for 1 month after ablation. The results showed that colchicine use both before and after the procedure significantly reduced the likelihood of PAP [27]. A recent systematic review reported pericarditis rates of 5.3% among colchicine recipients, which was less than a third of the frequency in the placebo group (16.5%), demonstrating that colchicine reduces the likelihood of PAP [28]. Another study examined the protective effect of a routine postoperative colchicine regimen (0.6 mg twice daily for 14 days post-AF ablation) against acute pericarditis following high-power, short-duration AF ablation. The results showed that prophylactic colchicine was not associated with a significant reduction in the incidence of post-ablation chest pain, pericarditis, 30-day hospitalization, or emergency room visits [29]. The PAPER study similarly showed that prophylactic colchicine did not affect the incidence of post-ablation pericarditis and even increased gastrointestinal side effects in AF patients [19].

In some of the studies mentioned above, independent relationships were not examined, and some assessed a narrow set of variables. In the current study, we investigated the relationship between PAP and a broad range of variables, including sociodemographic, clinical, demographic, laboratory, and ablation-procedure-related data. The contradictory findings in previous studies, especially regarding the relationship between prophylactic treatment and PAP, are notable. In our study, treatment choice was not found to be associated with PAP. We can explain the association of prolonged AF duration, CTI ablation, and sleep apnea with the development of pericarditis after AF ablation from a broader perspective. Prolonged AF duration is linked to more extended electrical and structural remodeling in the atrial tissue [30]. This process leads to persistent fibrosis, inflammation, and cellular changes in the atrial walls, potentially triggering a more intense inflammatory response after ablation. Patients with prolonged AF may require more manipulation or energy intensity during the ablation, increasing the likelihood of affecting pericardial tissue. CTI ablation, widely used in the treatment of typical atrial flutter, creates an additional ablation area [31]. Since the CTI region is located close to the pericardium, ablation in this area may lead to increased pericardial inflammation. CTI ablation may also extend the procedure time and result in more extensive energy distribution, further enhancing the inflammatory response. The absence of this specific association in previous studies may be due to the insufficient investigation of this factor and the unavailability of data. Lastly, sleep apnea exerts pressure on atrial structures and has been associated with chronic inflammation and oxidative stress [32]. Patients with sleep apnea are prone to hemodynamic instability and inflammation [32], which could intensify the inflammatory processes after ablation. The influence of sleep apnea on inflammatory processes may contribute to the development of pericarditis after ablation. This association may not have been previously observed because sleep apnea has not always been thoroughly examined in AF patients.

Given the limited data on this topic and, particularly, the comprehensive dataset examined in the present study, we believe the present results and data provide strong evidence and show the necessity of more detailed studies to identify more specific risk factors contributing to PAP. Furthermore, these results offer a new perspective on risk assessment and complication management after AF ablation. For instance, patients with longer AF duration, sleep apnea, and CTI ablation should be followed closely. In fact, the utilization of CTI ablation can be re-considered among patients with sleep apnea or prolonged AF, possibly reducing PAP risks. Discussing practical approaches, such as screening for sleep apnea or adjusting ablation strategies, could indeed provide valuable insights into reducing the risk of pericarditis. Implementing routine screenings for sleep apnea may help identify patients at higher risk and allow for targeted interventions. Additionally, refining ablation techniques or optimizing the energy settings based on individual patient profiles could further mitigate the risk of complications. Exploring these strategies could enhance our understanding of pericarditis. The clinical implications of our results may contribute to the optimization of patient management and complication prevention by enabling personalized treatment approaches in patients undergoing AF ablation.

In the current study, both RF ablation and cryoablation were compared; paroxysmal, persistent, and long-persistent AF were analyzed; both definitive and suspected pericarditis cases were examined; and the impact of numerous variables on PAP was investigated, contributing new risk factors to the literature. However, several limitations must be considered when interpreting the results. This study has the inherent limitations of a limited number of center and retrospective studies, such as limited generalizability and lack of pre-planned data collection. Although this study is one of the few studies that has examined PAP with such a broad dataset, patient profiles may vary due to geographic or socioeconomic factors, which could affect the applicability of the results to a broader population. Additionally, outcomes may differ in other countries or centers in which different clinical practices and treatment protocols are used. Therefore, future studies involving larger, multicenter, and international cohorts would be valuable for validating these findings. Nonetheless, the recurrence rate, pericardial effusion information, and follow-up times could not be obtained for all the patients and were, therefore, excluded from this study. Inevitably, the low percentage of patients with PAP may have affected the reliability of statistical analyses when determining risk factors for PAP. The effects of ablation procedures other than RF and cryoballoon ablation on PAP were not investigated, as these were the only procedures performed at our center.

## 5. Conclusions

The detailed analyses of our study, based on a large dataset during a 3-year period, demonstrates that prolonged AF duration, CTI ablation, and sleep apnea are risk factors for PAP. The consideration of these risk factors in decision making and patient management has the potential to enhance the safety of AF ablation procedures. Inflammatory markers such as SII, NLR, and PLR were not found to be associated with PAP. Future studies should investigate the relationship between other inflammatory biomarkers (potentially tissue-specific markers) and PAP in greater detail, as this could improve comprehension of the pathophysiological mechanisms linking inflammation and PAP, thereby facilitating risk assessment and reducing PAP likelihood.

## Figures and Tables

**Table 1:** Summary of sociodemographic and clinical characteristics with regard to pericarditis.

	Overall (*n* = 231)	Pericarditis			*p*
		No (*n* = 195)	Suspicious (*n* = 22)	Yes (*n* = 14)	
Age	58 (48–66)	58 (48–66)	57 (49–65)	54.5 (47–62)	0.692 ^‡^
Sex					
Female	112 (48.48%)	99 (50.77%)	4 (18.18%)	9 (64.29%)	**0.007 ^§^**
Male	119 (51.52%)	96 (49.23%)	18 (81.82%) *	5 (35.71%) ^#^	
Body mass index, kg/m^2^	27.72 (24.91–31.02)	27.60 (24.84–31.10)	27.49 (25.70–32.00)	28.65 (26.90–29.41)	0.816 ^‡^
Diabetes mellitus	33 (14.29%)	30 (15.38%)	1 (4.55%)	2 (14.29%)	0.450 ^¶^
Cerebrovascular disease	9 (3.90%)	7 (3.59%)	2 (9.09%)	0 (0.00%)	0.289 ^¶^
Hypertension	104 (45.02%)	91 (46.67%)	7 (31.82%)	6 (42.86%)	0.409 ^§^
Heart failure	28 (12.12%)	23 (11.79%)	2 (9.09%)	3 (21.43%)	0.485 ^¶^
Coronary artery disease	59 (25.54%)	49 (25.13%)	9 (40.91%)	1 (7.14%)	0.073 ^§^
CHA_2_DS_2_-VASc score	2 (1–3)	2 (1–3)	1 (0–3)	1 (1–2)	0.478 ^‡^
Sleep apnea	13 (5.63%)	7 (3.59%)	5 (22.73%) *	1 (7.14%)	**0.004 ^¶^**
Smoking status					
Non-smoker	123 (53.25%)	106 (54.36%)	9 (40.91%)	8 (57.14%)	0.752 ^¶^
Ex-smoker	47 (20.35%)	39 (20.00%)	5 (22.73%)	3 (21.43%)	
Active smoker	61 (26.41%)	50 (25.64%)	8 (36.36%)	3 (21.43%)	

The descriptive statistics are presented using mean ± standard deviation for normally distributed continuous variables, median (25th percentile–75th percentile) for non-normally distributed continuous variables, and frequency (percentage) for categorical variables. ^‡^ Kruskal–Wallis test, ^§^ chi-square test, ^¶^ Fisher–Freeman–Halton test, * significantly different from “No pericarditis” group, ^#^ significantly different from “Suspicious” group.

**Table 2:** AF-related and management data with regard to pericarditis.

	Overall (*n* = 231)	Pericarditis			*p*
		No (*n* = 195)	Suspicious (*n* = 22)	Yes (*n* = 14)	
Duration of AF					
Paroxysmal	98 (42.42%)	84 (43.08%)	13 (59.09%)	1 (7.14%) *^#^	**0.007 ^¶^**
Persistent	87 (37.66%)	76 (38.97%)	5 (22.73%)	6 (42.86%)	
Long persistent	46 (19.91%)	35 (17.95%)	4 (18.18%)	7 (50.00%) *	
Duration of AF, months	6 (1–12)	6 (0.75–11)	3.5 (1.5–11)	24 (12–36) *^#^	**<0.001 ^‡^**
Oral anticoagulant use					
None	0 (0.00%)	0 (0.00%)	0 (0.00%)	0 (0.00%)	0.219 ^¶^
Apixaban	129 (55.84%)	104 (53.33%)	15 (68.18%)	10 (71.43%)	
Edoxaban	28 (12.12%)	23 (11.79%)	4 (18.18%)	1 (7.14%)	
Rivaroxaban	68 (29.44%)	63 (32.31%)	3 (13.64%)	2 (14.29%)	
Warfarin	6 (2.60%)	5 (2.56%)	0 (0.00%)	1 (7.14%)	
Antiarrhythmic agent use					
None	5 (2.16%)	5 (2.56%)	0 (0.00%)	0 (0.00%)	0.063 ^¶^
Amiodarone	110 (47.62%)	89 (45.64%)	9 (40.91%)	12 (85.71%)	
Propafenone	113 (48.92%)	99 (50.77%)	12 (54.55%)	2 (14.29%)	
Sotalol	3 (1.30%)	2 (1.03%)	1 (4.55%)	0 (0.00%)	
Statin use	68 (29.44%)	55 (28.21%)	10 (45.45%)	3 (21.43%)	0.193 ^§^
Ejection fraction	60 (55–61)	60 (55–62)	60 (55–61)	59.5 (55–60)	0.247 ^‡^
Left-atrium diameter	41 (38–44)	40 (38–44)	39.5 (38–44)	44 (43–47) *^#^	**0.008 ^‡^**

The descriptive statistics are presented using mean ± standard deviation for normally distributed continuous variables, median (25th percentile–75th percentile) for non-normally distributed continuous variables, and frequency (percentage) for categorical variables. ^‡^ Kruskal–Wallis test, ^§^ chi-square test, ^¶^ Fisher–Freeman–Halton test, * significantly different from “No pericarditis” group, ^#^ significantly different from “Suspicious” group. Abbreviations: AF, atrial fibrillation.

**Table 3:** Ablation-related characteristics and pericarditis treatment, compared across pericarditis groups.

	Overall (*n* = 231)	Pericarditis			*p*
		No (*n* = 195)	Suspicious (*n* = 22)	Yes (*n* = 14)	
Redo AF ablation	15 (6.49%)	9 (4.62%)	3 (13.64%)	3 (21.43%) *	0.020 ^¶^
Type of ablation					
Radiofrequency ablation	130 (56.28%)	106 (54.36%)	11 (50.00%)	13 (92.86%) *^#^	0.016 ^§^
Cryoablation	101 (43.72%)	89 (45.64%)	11 (50.00%)	1 (7.14%)	
Number of RF applications	133.5 (96–154)	131 (94–154)	112 (81–142)	153 (143–174) *^#^	0.007 ^‡^
RF duration, sec	11.55 (7.8–13.2)	11.3 (7.7–12.8)	11.4 (8.7–14.4)	13.1 (11.9–14.2) *	0.013 ^‡^
Anterior watt	40 (40–50)	40 (40–50)	40 (35–45)	40 (35–40) *	0.015 ^‡^
Posterior watt	30 (30–50)	30 (30–50)	30 (30–35)	30 (30–30) *	0.035 ^‡^
Atrial scar					
Stage 0	36 (27.69%)	32 (30.19%)	3 (27.27%)	1 (7.69%)	0.227 ^¶^
Stage 1	73 (56.15%)	60 (56.60%)	6 (54.55%)	7 (53.85%)	
Stage 2	16 (12.31%)	10 (9.43%)	2 (18.18%)	4 (30.77%)	
Stage 3	3 (2.31%)	2 (1.89%)	0 (0.00%)	1 (7.69%)	
Stage 4	2 (1.54%)	2 (1.89%)	0 (0.00%)	0 (0.00%)	
Cryoablation duration, sec	1140 (1020–1220)	1080 (1020–1200)	1220 (1080–1280)	1500 (1500–1500)	0.058 ^‡^
Peak cryoablation temperature	−52 (−53–−50)	−52 (−53–−50)	−52 (−55–−50)	−51 (−51–−51)	0.781 ^‡^
Number of cryoablation applications	6 (5–6)	6 (5–6)	6 (5–7)	8 (8–8)	0.051 ^‡^
CTI ablation	45 (19.48%)	31 (15.90%)	5 (22.73%)	9 (64.29%) *^#^	<0.001 ^¶^
Ablation line	8 (3.46%)	5 (2.56%)	1 (4.55%)	2 (14.29%)	0.071 ^¶^
Anterior mitral	4 (1.73%)	3 (1.54%)	1 (4.55%)	0 (0.00%)	0.061 ^¶^
Roof	2 (0.87%)	1 (0.51%)	0 (0.00%)	1 (7.14%)	
Anterior mitral + roof	2 (0.87%)	1 (0.51%)	0 (0.00%)	1 (7.14%)	
Posterior-wall ablation	14 (6.06%)	8 (4.10%)	2 (9.09%)	4 (28.57%) *	0.004 ^¶^
Focal ablation	18 (7.79%)	17 (8.72%)	1 (4.55%)	0 (0.00%)	0.762 ^¶^
Treatment					
None	200 (86.58%)	195 (100.00%)	5 (22.73%)*	0 (0.00%) *	<0.001 ^¶^
Ibuprofen	9 (3.90%)	0 (0.00%)	9 (40.91%)*	0 (0.00%) ^#^	
Colchicine	16 (6.93%)	0 (0.00%)	6 (27.27%)*	10 (71.43%) *^#^	
Colchicine + ibuprofen	6 (2.60%)	0 (0.00%)	2 (9.09%)*	4 (28.57%) *	

The descriptive statistics are presented using mean ± standard deviation for normally distributed continuous variables, median (25th percentile–75th percentile) for non-normally distributed continuous variables, and frequency (percentage) for categorical variables. ^‡^ Kruskal–Wallis test, ^§^ chi-square test, ^¶^ Fisher–Freeman–Halton test, * significantly different from “No pericarditis” group, ^#^ significantly different from “Suspicious” group. Abbreviations: AF, atrial fibrillation; CTI, cavotricuspid isthmus; RF, radiofrequency.

**Table 4:** Summary of laboratory findings with regard to pericarditis groups.

	Overall (*n* = 231)	Pericarditis			*p*
		No (*n* = 195)	Suspicious (*n* = 22)	Yes (*n* = 14)	
Creatinine, mg/dL	0.90 (0.79–1.01)	0.88 (0.78–1.00)	0.93 (0.87–1.10)	0.86 (0.70–1.02)	0.080 ^‡^
Hemoglobin g/dL	13.70 ± 1.63	13.65 ± 1.70	14.01 ± 0.98	13.84 ± 1.40	0.579 ^†^
WBC (×10^3^)	7.10 (6.24–8.90)	7.10 (6.24–8.70)	7.85 (6.78–8.95)	7.92 (7.10–9.10)	0.087 ^‡^
Neutrophil (×10^3^)	4.40 (3.60–5.34)	4.26 (3.60–5.20)	4.88 (3.85–6.00)	4.55 (3.80–5.60)	0.302 ^‡^
Lymphocyte (×10^3^)	2.20 (1.80–2.70)	2.20 (1.80–2.62)	2.07 (1.80–2.40)	2.45 (2.20–2.92) *^#^	0.038 ^‡^
Platelet (×10^3^)	246 (214–270)	250 (216–270)	221.5 (198–246)	267 (235–290) ^#^	0.028 ^‡^
NLR	2.05 (1.57–2.57)	2.00 (1.57–2.57)	2.38 (1.86–3.17)	1.74 (1.45–2.24)	0.122 ^‡^
PLR	111.41 (89.29–146.96)	111.90 (89.58–147.78)	102.14 (83.00–162.86)	111.88 (66.67–120.45)	0.435 ^‡^
SII (×10^3^)	494.45 (355.63–657.82)	494.45 (343.03–660.26)	472.79 (390.48–905.67)	505.60 (441.90–566.14)	0.836 ^‡^
Glucose, mg/dL	96 (89–106)	97 (89–106)	97 (89–105)	95.5 (89–104)	0.943 ^‡^
Albumin, g/dL	4.22 ± 0.33	4.22 ± 0.34	4.20 ± 0.25	4.22 ± 0.32	0.945 ^†^
Total cholesterol, mg/dL	188 (161–213)	187 (160–213)	188.5 (161–225)	190.5 (167–202)	0.933 ^‡^
HDL-C, mg/dL	38 (33–43)	38 (33–43)	37.5 (31–42)	35 (31–43)	0.464 ^‡^
LDL-C, mg/dL	112.75 ± 34.26	112.30 ± 34.41	112.01 ± 35.13	120.16 ± 32.25	0.707 ^†^
Triglyceride, mg/dL	162 (111–208)	165 (111–208)	157 (105–214)	152 (99–193)	0.961 ^‡^
GGT, U/L	17 (12–26)	17 (12–26)	15.5 (11–37)	15.5 (12–26)	0.991 ^‡^
CRP, mg/L	2.1 (1.0–3.4)	2.1 (1.0–3.4)	2.1 (1.1–3.2)	2.95 (0.8–4.0)	0.772 ^‡^

The descriptive statistics are presented using mean ± standard deviation for normally distributed continuous variables and median (25th percentile–75th percentile) for non-normally distributed continuous variables. ^†^ One-way analysis of variance (ANOVA), ^‡^ Kruskal–Wallis test, * significantly different from “No pericarditis” group, ^#^ significantly different from “Suspicious” group. Abbreviations: CRP, C-reactive protein; GGT, gamma-glutamyl transferase; HDL-C, high-density lipoprotein cholesterol; LDL-C, low-density lipoprotein cholesterol; NLR, neutrophil-to-lymphocyte ratio; PLR, platelet-to-lymphocyte ratio; SII, systemic immune-inflammation index; WBC, white blood cell.

**Table 5:** Significant factors independently associated with definite post-ablation pericarditis, multivariable logistic regression.

	β Coefficient	Standard Error	*p*	Exp(β)	95% CI for Exp(β)	
Duration of AF, months	0.017	0.007	0.026	1.017	1.002	1.032
CTI ablation	2.056	0.601	0.001	7.812	2.404	25.386
Constant	−3.837	0.494	<0.001	0.022		

Nagelkerke R^2^ = 0.210. Abbreviations: AF, atrial fibrillation; CI, confidence interval; CTI, cavotricuspid isthmus.

**Table 6:** Significant factors independently associated with suspected or definitive post-ablation pericarditis, multivariable logistic regression.

	β Coefficient	Standard Error	*p*	Exp(β)	95% CI for Exp(β)	
Sleep apnea	1.622	0.611	0.008	5.062	1.529	16.765
CTI ablation	1.181	0.404	0.003	3.256	1.476	7.186
Constant	−2.140	0.241	<0.001	0.118		

Nagelkerke R^2^ = 0.111. Abbreviations: CI, confidence interval; CTI, cavotricuspid isthmus.

## Data Availability

The data that support the findings of this study are available from the corresponding author upon request.
